# Chytrid parasitism facilitates trophic transfer between bloom-forming cyanobacteria and zooplankton (*Daphnia*)

**DOI:** 10.1038/srep35039

**Published:** 2016-10-13

**Authors:** Ramsy Agha, Manja Saebelfeld, Christin Manthey, Thomas Rohrlack, Justyna Wolinska

**Affiliations:** 1 Leibniz-Institute of Freshwater Ecology and Inland Fisheries (IGB), Berlin, Germany; 2 Department of Biology, Chemistry, Pharmacy, Institute of Biology, Freie Universität, Berlin (FU), Germany; 3 Norwegian University of Life Sciences (NMBU), Department of Environmental Sciences, Ås, Norway

## Abstract

Parasites are rarely included in food web studies, although they can strongly alter trophic interactions. In aquatic ecosystems, poorly grazed cyanobacteria often dominate phytoplankton communities, leading to the decoupling of primary and secondary production. Here, we addressed the interface between predator-prey and host-parasite interactions by conducting a life-table experiment, in which four *Daphnia galeata* genotypes were maintained on quantitatively comparable diets consisting of healthy cyanobacteria or cyanobacteria infected by a fungal (chytrid) parasite. In four out of five fitness parameters, at least one *Daphnia* genotype performed better on parasitised cyanobacteria than in the absence of infection. Further treatments consisting of purified chytrid zoospores and heterotrophic bacteria suspensions established the causes of improved fitness. First, *Daphnia* feed on chytrid zoospores which trophically upgrade cyanobacterial carbon. Second, an increase in heterotrophic bacterial biomass, promoted by cyanobacterial decay, provides an additional food source for *Daphnia*. In addition, chytrid infection induces fragmentation of cyanobacterial filaments, which could render cyanobacteria more edible. Our results demonstrate that chytrid parasitism can sustain zooplankton under cyanobacterial bloom conditions, and exemplify the potential of parasites to alter interactions between trophic levels.

Trophic interactions govern the flow of material and energy in ecosystems and modulate many of their fundamental properties, such as productivity, regime shifts, or biogeochemical cycles^1^,^2^,^3^. Advances in food web theory and modelling have contributed to our picture of the network of feeding relationships in ecological communities. Still, they often fail to explain processes observed in natural systems^4^. One reason for this is that most food web studies do not incorporate what is perhaps the most common trophic interaction - parasitism^5^. Despite their ubiquity, parasites are usually overlooked because of their cryptic nature, the difficulties in quantifying their effects, and their assumed low biomass^6^. However, they can account for greater biomass than predators^7^ and participate in the majority of trophic links^8^. Parasites can modulate trophic flows in a number of ways. They can drive reductions in host biomass, not only by increasing host mortality rates, but also by influencing growth, fecundity, nutritional status, susceptibility to predation, or behaviour^9^. While their role as consumers is better known, parasites can also be prey for other organisms. They can be consumed together with their host (i.e. concomitant predation) or as free living life stages. Given the enormous reproductive output of parasites, free living infecting stages potentially constitute a significant nutrient source and can account for a substantial transfer of material and energy to higher trophic levels^10^,^11^.

The efficiency of energy and material entry into the food web is largely determined by the trophic coupling between primary and secondary production. In aquatic pelagic ecosystems, primary production is often dominated by cyanobacteria. Promoted by eutrophication and global warming^12^,^13^, cyanobacteria often develop into blooms that severely disrupt ecosystem functioning and raise health concerns due to the production of diverse toxins^14^,^15^. Cyanobacteria display high resistance to grazing, which often leads to the decoupling of primary and secondary production and inefficient carbon transfer to zooplankton^16^. The inability of zooplankton to exert effective top-down control on cyanobacterial populations has traditionally been linked to the poor edibility of cyanobacteria with colonial or filamentous morphologies, the production of toxic metabolites, and their low nutritional value^17^. Meta-analyses of experimental data have shown that, although grazing resistance cannot be generalised, cyanobacteria in fact constitute a poor food resource, as they lack essential nutritional compounds for zooplankton, such as sterols and polyunsaturated fatty acids (PUFAs)^18^,^19^. However, field observations often report a high biomass of grazers during bloom events^20^,^21^,^22^, suggesting alternative sources of nutrition capable of sustaining zooplankton growth.

Besides grazing, parasitism can act as an additional top-down control on phytoplankton^23^,^24^. In addition to virus and prokaryotic parasites (e.g. lytic bacteria), a so-far hidden diversity of small microeukaryotes has been revealed by metagenomics surveys, many of which display parasitic lifestyles^25^. Among these eukaryotic parasites, phytoplankton is particularly affected by chytrids, a group of primitive fungi characterised by a free-swimming zoosporic life stage^26^. Chytrid infection is lethal and has the potential to act as a controlling agent on cyanobacterial populations, often reaching epidemic proportions^24^. Zoosporic fungi can be an important food source for zooplankton, as they provide sterols and long-chain PUFAs that are lacking in prokaryotic prey^27^,^28^,^29^. Arthropods cannot synthesise these compounds *de novo*, so that they need to obtain these essential lipids from their diet^30^,^31^. Experimental work has shown that the cladoceran *Daphnia*, a keystone crustacean herbivore that drives much of the secondary production in pelagic ecosystems^32^, is able to feed on chytrid zoospores infecting the large inedible diatom *Asterionella formosa*. Thus, constituents may potentially be channeled from inedible algae to zooplankton *via* a trophic link termed the “mycoloop”^33^,^34^. In a similar way, chytrids may enable nutrient transfer from *A. formosa* to copepods^35^. However, due to its size, *A. formosa* cannot be grazed upon either by *Daphnia* or copepods, leaving open the question of whether chytrids can enhance the coupling between zooplankton and smaller, yet nutritionally suboptimal phytoplankton, like cyanobacteria.

Here, we experimentally address the interrelation between predator-prey and host-parasite interactions. To do so, we used a host-parasite system based on the filamentous, bloom-forming cyanobacterium *Planktothrix agardhii* and its obligate chytrid parasite *Rhizophydium megarrhizum*. A laboratory experiment was conducted in which four genotypes of *Daphnia galeata* were maintained under quantitatively comparable diets of infected and uninfected cyanobacteria, together with additional experimental treatments consisting of chytrid zoospores and heterotrophic bacteria suspensions. We hypothesise that chytrid infection on phytoplankton can sustain zooplankton growth and improve its fitness under cyanobacterial dominance.

## Results

**Figure 1:**
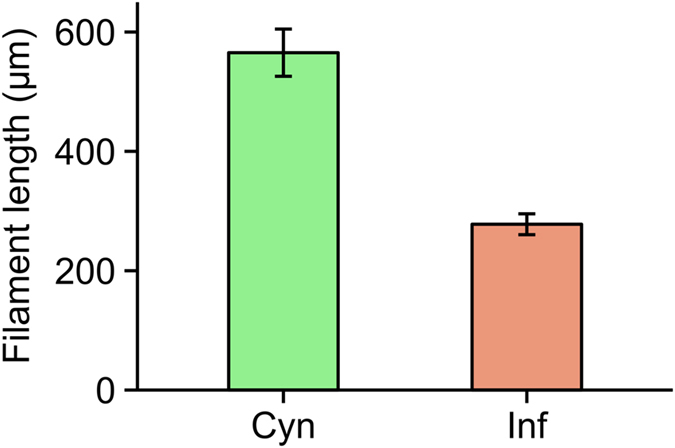
Mean length of cyanobacteria filaments. Data are shown as mean filament lengths (±s.e.m.) in each feeding suspension (n = 11). Kruskal-Wallis test revealed significant differences between treatments (χ^2^ = 13.772, p = 0.0002) Cyn: uninfected cyanobacteria, Inf: infected cyanobacteria.

Table 1 shows particulate organic carbon (POC) concentrations and counts of cyanobacteria, zoospores and heterotrophic bacteria in the respective treatments. In both infected and uninfected cyanobacteria treatments, POC concentrations were well above *Daphnia* requirements (>0.6 mg C l^−1^; refs 36,37), although about 20% lower in the infected compared to the uninfected treatment. Additional treatments consisting of zoospores or heterotrophic bacteria provided comparable food quantities, yet supplied only about 10% of POC relative to cyanobacteria treatments. The density of heterotrophic bacteria in the uninfected cyanobacteria treatment was about 80% lower compared to other treatments. Infected cyanobacterial filaments were halved in length relative to conditions of absence of infection (Fig. 1).

Two-way ANOVAs showed a significant effect of diet (i.e. treatment) on all measured fitness parameters. *Daphnia* genotype had an effect for all parameters except fecundity (Table 2). Significant treatment by genotype interactions were found for fecundity and body size of *Daphnia*, both of adults and offspring. For the parameters age at maturity and body size of adults (which presented non-normal distributions), additional two-way ANOVAs were performed on aligned rank transformed data resulting in the same significance levels for adults body size, and an additional significant treatment by genotype interaction for age at maturity (p < 0.001).

**Figure 2:**
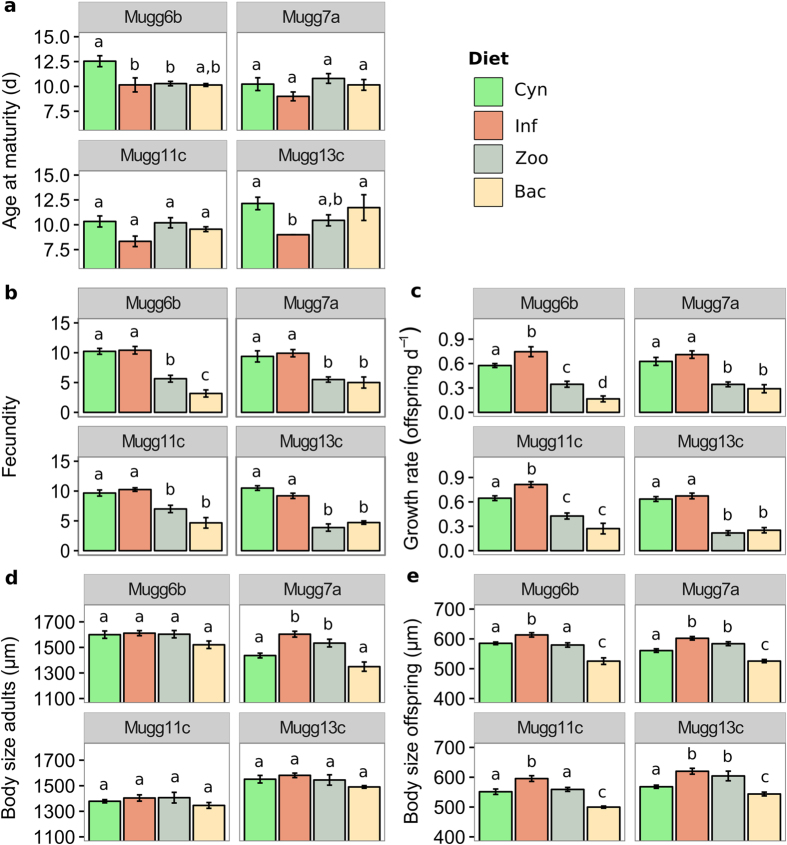
Fitness parameters of *Daphnia* genotypes fed with different diets. (**a**) Age at maturity, (**b**) fecundity, (**c**) growth rate, (**d**) body size of adults, (**e**) body size of offspring. Data are shown as means (±s.e.m.). Significant differences between treatments within genotypes (Mugg6b, Mugg7a, Mugg11c, Mugg13c) are indicated by different lowercase letters. Cyn: uninfected cyanobacteria, Inf: infected cyanobacteria, Zoo: zoospores, Bac: heterotrophic bacteria. The respective *n* are shown in Table 2.

Within-genotype comparisons revealed that, for all fitness parameters except fecundity, at least one *Daphnia* genotype performed better on an infected compared to an uninfected cyanobacteria diet (Fig. 2, Table 2). Two genotypes matured significantly earlier (Fig. 2a) and larger body sizes in adults were found for one genotype (Fig. 2d). All genotypes displayed offspring with bigger size (Fig. 2e), whereas two genotypes showed higher growth rates when fed with infected cyanobacteria (Fig. 2c). There were no significant differences in total offspring number (Fig. 2b) or in the proportion of individuals that survived until the third reproductive cycle between infected and uninfected cyanobacteria treatments (data not shown).

**Figure 3:**
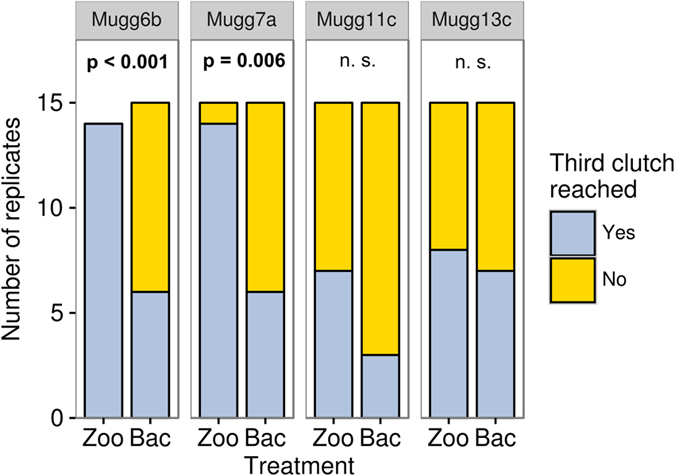
Number of replicates of each *Daphnia* genotype that reached the third reproductive cycle when fed with zoospores (Zoo) and heterotrophic bacteria (Bac) suspensions. Significant differences are highlighted by p-values in bold (Fisher’s exact test) (n.s.: not significant). Comparisons of infected and uninfected cyanobacteria treatments yielded no significant differences (data not shown).

Zoospore and heterotrophic bacteria diets induced a strong reduction in total offspring number and growth rates relative to treatments supplying cyanobacteria (Fig. 2b,c, Table 2). However, despite lower POC concentrations supplied (about 90% lower), *Daphnia* on a zoospore diet performed equal or better in all other fitness parameters compared to the uninfected cyanobacteria treatment. In all cases, *Daphnia* displayed bigger offspring on zoospore *vs.* bacteria (Fig. 2e). On a zoospore diet significant differences in size of adults, total offspring and growth rates were found for one out of four genotypes when compared with a heterotrophic bacteria diet. Also, a higher proportion of replicates survived until the third reproductive cycle when *Daphnia* were fed with zoospores (Fig. 3), although food quantity in both treatments was comparable (Table 1).

## Discussion

In aquatic pelagic systems, primary and secondary production is subject to severe decoupling when poorly edible colonial or filamentous cyanobacteria dominate primary production^16^. Here, we addressed the question of whether parasites are able to prevent such decoupling by providing alternative trophic links between cyanobacteria and zooplankton. Chytrid parasites of cyanobacteria, although typically neglected, are ubiquitous and often burst into epidemics, reaching infection prevalence over 90%^38^,^39^. Our study shows that *Daphnia* fitness can be significantly improved when cyanobacteria are attacked by parasites. Upon chytrid infection, filamentous cyanobacteria get fragmented, potentially increasing their edibility, and alternative food sources are made available for grazers in the form of chytrid zoospores and increased abundances of heterotrophic bacteria.

In our experiment, we observed for at least one genotype a significant improvement in fitness in terms of age at maturity, growth rates, and body size of adults and offspring when *Daphnia* was fed with infected cyanobacteria diet compared to an uninfected cyanobacteria diet. We measured five different parameters to better characterize *Daphnia* fitness. Under natural conditions, earlier born offspring can imply strong contributions to the establishment of the population, especially under food limitation and in the presence of predators^40^. Body size is another important fitness trait in *Daphnia*. Bigger size at birth leads to higher resistance to starvation and larger body size of adults^41^,^42^. Adult size in turn is positively correlated with clutch size^42^,^43^; hence, larger animals potentially contribute to the population with higher amounts of offspring. In our study, no differences between infected and uninfected diets were detected concerning total number of offspring. However, in natural settings, the time needed to produce offspring can be another crucial factor. We thus compared growth rates between genotypes. Two genotypes displayed increased growth rates in the infected diet compared to healthy cyanobacteria, indicating that an infected cyanobacteria diet contributes to *Daphnia* fitness by accelerating reproduction, rather than increasing total offspring.

To disentangle the contribution of the aforementioned additional food sources to *Daphnia* fitness, zoospores and heterotrophic bacteria were separated from the infected cultures and used as food in two additional experimental treatments. The resulting reduction in POC in these treatments (about 90%) compared to cyanobacterial diets induced a strong reduction in the production of offspring in all genotypes. However, these adverse effects were not observed in other fitness parameters. Notably, the zoospore diet yielded higher or otherwise not significantly different fitness in other parameters compared to the uninfected cyanobacterial diet, despite a 90% reduction in supplied POC. This strongly suggests enhanced carbon transfer efficiency when conveyed to grazers *via* chytrid zoospores. Zoospores are within the optimal size range for ingestion by *Daphnia*, and display high cellular contents of PUFAs and sterols (in particular, cholesterol and its precursors sitosterol and stigmasterol^34^,^44^) that prevent the lipid limitation typically observed when cladoceran grazers feed on cyanobacteria^45^. By extracting, transforming and repacking nutrients from cyanobacteria into higher quality, more readily ingestible zoospores, chytrids upgrade cyanobacterial carbon for zooplankton, thereby increasing the coupling between primary and secondary production under cyanobacterial dominance or bloom conditions.

A chytrid-mediated trophic link between *Daphnia* and inedible diatoms has been proposed previously, giving rise to the concept of the “mycoloop”. Kagami *et al.*^34^ undertook a 6-day life-table experiment in which *Daphnia* were fed with chytrid-infected and uninfected cultures of the large inedible diatom *Asterionella formosa. Daphnia* displayed significantly larger body size when fed with infected diatoms, as a result of feeding on chytrid zoospores. However, in that study reproduction was not assessed, raising questions as to whether zoospores alone can sustain *Daphnia* populations. The present experiment was conducted over three reproductive cycles, which not only allowed testing for population viability, but also minimised the potential effects of maternal nutrient reserves, which might mask nutrient limitations imposed by the different diets tested^46^. Moreover, in the former study, limitations in the experimental design (6 replicates per treatment with only one replicate left in the uninfected diet treatment) complicate the interpretation of the results. Whereas that study tested only a single *Daphnia* genotype, the present experiment included four different genotypes. Our results showed significant effects of genotype and/or genotype by diet interactions for all measured fitness parameters and support the importance of addressing inter-clonal variability in *Daphnia* populations^47^,^48^. Finally, whereas *Asterionella* represents an inaccessible carbon source that cannot be exploited by *Daphnia,* cyanobacteria constitute a suboptimal, yet edible food source. This difference uncovers a new facet of the mycoloop whereby chytrid parasites act as trophic upgraders of suboptimal prey, illustrating their potential to operate in ways other than making carbon available from inedible sources.

Our experiment shows that chytrid zoospores alone are sufficient to sustain not only *Daphnia* growth, but also reproduction, and can enhance fitness relative to parasite-free conditions. This demonstrates that natural zooplankton populations can, in principle, be sustained solely by mycoloop contributions. The extent of the mycoloop largely depends on the range of naturally-occurring zoospore densities in the water column. Direct quantifications of zoospores from environmental samples, although seldom undertaken, have shown maximal zoospore densities of up to 500 zoospores ml^−1 ^ treatments^49^, which are about one quarter of those provided in our experimental treatments. However, much higher densities can potentially be reached. For example, Rasconi *et al.*^39^ reported sporangia (i.e. sessile reproductive structures that release new zoospores upon maturation) densities of 3 × 10^4^ ml^−1^ under epidemic conditions. Considering a mean release of 4-25 zoospores per sporangium^50^, free-swimming zoospores could reach densities two or even three orders of magnitude higher than provided in our assay, suggesting that the contribution of zoospores to zooplankton diet under natural conditions may be greater than shown by this experiment.

In addition to chytrid zoospores, our results suggest that heterotrophic bacteria cannot be neglected as an extra food source for *Daphnia.* In contrast to the study of Kagami *et al.*^34^, chytrid infection caused a 5-fold increase in bacterial densities compared to uninfected conditions. Such increase is attributable to the decay of infected cyanobacteria and the subsequent release of dissolved organic carbon, which can be readily used by heterotrophic bacteria for growth. Observed increase in bacterial biomass was enough to sustain *Daphnia* growth and reproduction. However, on a purified bacterial diet, fewer individuals reached the third reproductive cycle (Fig. 3), and their offspring were smaller relative to the zoospore diet in all cases (Fig. 2e). This indicates that bacteria constitute a food source of lower quality than chytrids, which is consistent with the general lack of essential lipids in prokaryotes^51^−^53^. Still, increased shares of bacterial food sources during the decay of inedible phytoplankton can act as a conveyor of dietary energy to *Daphnia.* Bacteria repack otherwise poorly ingestible cyanobacteria into smaller, easily ingestible particles and potentially detoxify cyanobacterial carbon. Moreover, bacterial proliferation under natural conditions activates the microbial loop^54^, promoting the growth of heterotrophic nanoflagellates and ciliates, which in turn have the potential to upgrade the biochemical quality of prokaryotic carbon by *de novo* synthesis of essential lipids^55^,^56^. Thus, increased bacterial densities may have more profound effects under natural conditions than our experiment can show.

Beside the aforementioned effects of zoospores and increased densities of heterotrophic bacteria, cyanobacterial filaments underwent fragmentation upon chytrid infection. Although the present experimental design did not allow a direct comparison of *Daphnia* grazing rates on shorter (infected) and longer (uninfected) filaments, a halving in length arguably reduces mechanical feeding interference and could facilitate grazing^20^. Filament fragmentation upon infection by chytrids has been documented in nature^57^, but its implications for zooplankton grazing remain to be assessed.

All in all, our experiment shows how the decoupling of primary and secondary production, typically assumed in cyanobacteria-dominated aquatic ecosystems, can be circumvented by the effect of parasites, which can establish new, alternative trophic links and enhance existing ones, facilitating the transfer of carbon up the food web.

## Methods

### *Daphnia,* cyanobacteria and chytrid cultures

Four *D. galeata* genotypes (Mugg6b, Mugg7a, Mugg11c, Mugg13c) were isolated from the eutrophic Lake Müggelsee in eastern Germany. Clonal lines were kept in jars containing 200 ml of medium (five individuals per jar) consisting of a mixture of 95% synthetic *Daphnia* medium (based on ultrapure water, trace elements and phosphate buffer) and 5% Z8 medium^58^. Pre-experimental conditions included: constant temperature of 20 ± 1 °C, 12:12 h light:dark cycle, and feeding every two days with 1 mg C l^−1^ of the green algae *Scenedesmus obliquus*. The filamentous *Planktothrix agardhii* strain NIVA CYA630, isolated from Lake Lyseren (Norway), was maintained in Z8 medium as non-axenic semi continuous cultures under 20 °C and 20 μmol photons m^−2^ s^−1^. The obligate chytrid parasite strain Chy-Kol2008, isolated from Lake Kolbotnvatnet (Norway) and identified as *Rhizophydium megarrhizum*^59^, was used to infect cultures of the cyanobacterial strain NIVA CYA630.

### Preparation and characterisation of feeding treatments

*Daphnia* were fed with two diets consisting of uninfected or chytrid-infected cyanobacteria (*Planktothrix agardhii* NIVA CYA630), respectively, both providing similar POC concentrations above *Daphnia* requirements (Table 1). To determine feeding volumes, optical density at 750 nm (measured with 5 cm cuvettes) was correlated with POC concentrations. For the preparation of chytrid-infected cyanobacterial feeding suspensions, a standard infection protocol was used: seven days before each feeding occasion, chytrid zoospores (final conc. 2000 ml^−1^) were added to exponentially growing cyanobacterial cultures (density 4 × 10^5^ filaments ml^−1^) and incubated at 20 °C and 20 μmol photons m^−2^ s^−1^. After incubation, infected cultures were used to feed *Daphnia* providing a final POC concentration of ~1 μg C ml^−1^. Adequate feeding volumes were estimated by independent POC determinations from five standard infected replicate cultures before the start of the experiment.

In order to disentangle the contribution of chytrid zoospores and heterotrophic bacteria to changes in *Daphnia* fitness under infected and uninfected cyanobacterial diets, two additional feeding treatments were included, consisting of chytrid zoospores (and heterotrophic bacteria) and heterotrophic bacteria only, respectively. At each feeding occasion, zoospore suspensions were obtained by sequential filtration of the cultures used for the infected cyanobacterial treatment through a 10 μm nylon mesh, followed by 5 and 3 μm polycarbonate filters. The filtrate was microscopically checked for the absence of cyanobacterial filaments and used as a zoospore feeding suspension. The remaining volume was filtered through a 1 μm filter. The zoospore-free filtrate was microscopically checked for the absence of zoospores and used as a heterotrophic bacteria feeding suspension. When feeding with zoospores and heterotrophic bacteria, the same feeding volume as for the infected cyanobacteria diet was used.

For all four treatments and feeding occasions, acid Lugol and formaldehyde-fixed (2% final concentration) samples were collected. Cyanobacteria and zoospore densities were determined by Utermöhl’s technique^60^. Heterotrophic bacteria were counted in a haemocytometer under an epifluorescence microscope after staining with 4′, 6-diamidino-2-phenylindole (DAPI; 1 μg ml^−1^ final conc.). For each feeding occasion, the lengths of at least fifty *Planktothrix* filaments in both infected and uninfected treatments were measured under a Nikon Ti Eclypse inverted microscope using the NIS-Element BR 4.5 software. Actual POC concentrations of all treatments at each feeding occasion (Table 1) were determined by filtering aliquots of each feeding suspension through pre-combusted and pre-weighted GF/F filters (pore size approx. 0.7 μm), dried for at least 24 h, weighed and analysed using an Elementar Vario EL analyzer. POC concentrations in heterotrophic bacteria suspensions were estimated from bacterial counts, assuming an average carbon content of 20 fg cell^−1^ 61.

### Experimental setup

*Daphnia* neonates of the 3^rd^ generation, all born within 24 h, were transferred individually to 50 ml vials containing 40 ml synthetic *Daphnia* medium. 15 replicates per treatment and genotype were set up. Clonal line identities were blinded. *Daphnia* were fed every 2 days with the appropriate diet (cyanobacteria, infected cyanobacteria, zoospores, heterotrophic bacteria) and media was exchanged every 4 days. All replicates were checked daily for survival and offspring production. Offspring were counted and frozen at −20 °C for subsequent analysis. Offspring found to be dead were excluded from body size, fecundity and growth rate analyses. Adult *Daphnia* were taken out of the experiment after the release of the 3^rd^ clutch and frozen. After 24 days, the experiment was stopped for all remaining replicates that had not reached the 3^rd^ clutch (n = 12) or had not reproduced at all (n = 3); in the latter case *Daphnia* were checked microscopically for the presence of males. Body size (length from top of head to base of tail spine) of offspring from the first clutch and of adults that had released three clutches was measured using a Nikon SMZ 25 stereomicroscope and NIS-Element BR 4.5 software.

### Statistical analysis

Three replicates were excluded from the overall data set: two that were found to be males and one that lay at the bottom of the vial for several days in the third week, barely moving, before recovering and reproducing toward the end of the experiment. The following *Daphnia* fitness parameters were assessed: maturity (age at first reproduction), fecundity (total number of living offspring), growth rate (number of living offspring per day), body size of adults and body size of offspring from the first clutch. To assess differences in age at maturity and offspring size, replicates that reproduced at least once were included in the analyses. For fecundity and growth rates, only replicates that released a third clutch were included in the analyses. If multiple offspring were born, body size of all living individuals was measured and averaged for the analysis. Occasionally, *Daphnia* individuals showed deformed bodies upon thawing; these individuals were excluded from body size analyses. Two-way ANOVAs were performed for each fitness parameter, including diet (i.e. treatment) and genotype as fixed factors, followed by a contrast test for the effect of diet within genotypes (least-squares means test with Holm’s p-value adjustment). Data of the parameters fecundity and body size of offspring were transformed (see Table 2). For two parameters (maturity and body size of adults), data were not normally distributed, even after transformation. Results shown in the main text stem from parametric ANOVAs, given that both parameters showed equal variances of the residuals and that ANOVA is reasonably robust to non-normality. However, in these two cases, a non-parametric two-way ANOVA on aligned rank transformed data^62^ was performed in parallel. The number of replicates per genotype that reached the third reproductive cycle (within the 24-day experiment) under each treatment was compared using Fisher´s exact test (uninfected *vs.* infected and zoospores *vs.* bacteria). All statistical analyses were performed in Rstudio (v.0.99.903).

## Additional Information

**How to cite this article**: Agha, R. *et al.* Chytrid parasitism facilitates trophic transfer between bloom-forming cyanobacteria and zooplankton (*Daphnia*). *Sci. Rep.* **6**, 35039; doi: 10.1038/srep35039 (2016).

## Figures and Tables

**Table 1:** Final carbon concentrations, and filament, zoospore and heterotrophic bacteria densities supplied to *Daphnia* in each feeding treatment.

Treatment	Carbon (mg l^−1^)	Filaments (ml^−1^)	Zoospores (ml^−1^)	Bacteria (10^6^ ml^−1^)
Cyn	1.01 ± 0.020	1,901 ± 100	—	0.75 ± 0.06
Inf	0.80 ± 0.090	1,582 ± 171	2,433 ± 285	4.42 ± 0.30
Zoo	0.09 ± 0.005	—	2,182 ± 263	4.45 ± 0.23
Bac	0.08 ± 0.004	—	—	4.24 ± 0.20

Cyn: uninfected cyanobacteria, Inf: infected cyanobacteria, Zoo: zoospores, Bac: heterotrophic bacteria. Data are shown as means (± s.e.m.) of all feeding occasions (*n* = 11).

**Table 2:** Effect of different food treatments on *Daphnia* fitness parameters.

Two-way ANOVA	Genotype	Contrast test						*n*			
Parameter		Cyn vs. Inf	Cyn vs. Zoo	Cyn vs. Bac	Inf vs. Zoo	Inf vs. Bac	Zoo vs. Bac	Cyn	Inf	Zoo	Bac
Age at maturity*											
* t* F_3,171_ = 9.73, **p** < **0.001** g F_3,171_=4.36, **p < 0.01*** t:g* F_9,171_ = 1.44, p = 0.175	Mugg6b	**0.019**	**0.023**	0.051	1.0	1.0	1.0	13	13	14	7
	Mugg7a	0.658	1.0	1.0	0.139	0.658	1.0	13	12	15	13
	Mugg11c	1.0	1.0	1.0	0.166	0.695	1.0	12	12	10	9
	Mugg13c	**<0.01**	0.207	0.612	0.369	**0.012**	0.369	14	10	9	11
Fecundity^1^											
* t* F_3,147_ = 94.19, **p < 0.001** g F_3,147_ = 0.92, p = 0.433* t:g* F_9,147_ = 2.67, **p < 0.01**	Mugg6b	0.869	**<0.001**	**<0.001**	**<0.001**	**<0.001**	**<0.01**	13	12	14	6
	Mugg7a	0.871	**<0.001**	**<0.001**	**<0.001**	**<0.001**	0.871	13	12	14	6
	Mugg11c	0.485	**0.017**	**<0.001**	**<0.01**	**<0.001**	0.085	12	12	7	3
	Mugg13c	0.306	**<0.001**	**<0.001**	**<0.001**	**<0.001**	0.306	14	10	8	7
Growth rate											
* t* F_3,147_ = 118.17, **p < 0.001** g F_3,147_ = 3.43, **p = 0.019*** t:g* F_9,147_ = 1.40, p = 0.192	Mugg6b	**<0.01**	**<0.001**	**<0.001**	**<0.001**	**<0.001**	**<0.01**	13	12	14	6
	Mugg7a	0.213	**<0.001**	**<0.001**	**<0.001**	**<0.001**	0.396	13	12	14	6
	Mugg11c	**<0.01**	**<0.01**	**<0.001**	**<0.001**	**<0.001**	0.084	12	12	7	3
	Mugg13c	0.953	**<0.001**	**<0.001**	**<0.001**	**<0.001**	0.953	14	10	8	7
Body size adults*											
* t* F_3,146_ = 9.15, **p < 0.001 4** g F_3,146_ = 38.72, **p < 0.001*** t:g* F_9,146_ = 2.09, **p** ** < 0.001**	Mugg6b	1.0	1.0	0.288	1.0	0.250	0.288	13	12	14	6
	Mugg7a	**<0.001**	**0.014**	0.093	0.093	**<0.001**	**<0.001**	13	12	14	6
	Mugg11c	1.0	1.0	1.0	1.0	1.0	1.0	12	12	7	3
	Mugg13c	1.0	1.0	0.703	1.0	0.259	0.943	14	9	8	7
Body size offspring^2^											
* t* F_3,143_ = 88.28, **p < 0.001 ** g F_3,143_ = 14.52, **p < 0.001*** t:g* F_9,143_ = 2.37, **p** **= 0.016**	Mugg6b	**0.036**	0.451	**<0.001**	**<0.01**	**<0.001**	**<0.001**	12	13	14	4
	Mugg7a	**<0.001**	**0.030**	**<0.001**	0.115	**<0.001**	**<0.001**	12	11	10	9
	Mugg11c	**<0.001**	0.290	**<0.001**	**<0.01**	**<0.001**	**<0.001**	10	12	8	5
	Mugg13c	**<0.001**	**0.011**	**<0.01**	0.236	**<0.001**	**<0.001**	13	9	7	10

Results of two-way ANOVAs (for effects of *t* treatment, *g* genotype and *t:g* treatment by genotype interaction) followed by contrast tests (comparisons between treatments within genotypes) are shown. The number of replicates (*n*) included in each comparison is given. Discrepancies in number of replicates between parameters resulted from unmeasurable animals (*see* Methods). Significant p-values are depicted in bold. Cyn: uninfected cyanobacteria, Inf: infected cyanobacteria, Zoo: zoospores, Bac: heterotrophic bacteria.

^*^data are not normally distributed but show equal variances of the residuals (*see* Methods).

^1^data^(1/2)^ transformed.

^2^data^(−4)^ transformed.
